# Intracoronary ECG ST-segment shift remission time during reactive myocardial hyperemia: a new method to assess hemodynamic coronary stenosis severity

**DOI:** 10.1152/ajpheart.00481.2024

**Published:** 2024-09-06

**Authors:** Marius Reto Bigler, Andrea Kieninger-Gräfitsch, Miklos Rohla, Noé Corpateaux, Frédéric Waldmann, Reto Wildhaber, Jonas Häner, Christian Seiler

**Affiliations:** ^1^Department of Cardiology, University Hospital Bern (Inselspital), University of Bern, Bern, Switzerland; ^2^Institute for Medical Engineering and Medical Informatics, University of Applied Sciences and Arts Northwestern Switzerland, Muttenz, Switzerland

**Keywords:** autonomous algorithm, coronary artery disease, fractional flow reserve, intracoronary electrocardiogram, myocardial ischemia

## Abstract

Fractional flow reserve (FFR) measurements are recommended for assessing hemodynamic coronary stenosis severity. Intracoronary ECG (icECG) is easily obtainable and highly sensitive in detecting myocardial ischemia due to its close vicinity to the myocardium. We hypothesized that the remission time of myocardial ischemia on icECG after a controlled coronary occlusion accurately detects hemodynamically relevant coronary stenosis. This retrospective, observational study included patients with chronic coronary syndrome undergoing hemodynamic coronary stenosis assessment immediately following a strictly 1-min proximal coronary artery balloon occlusion with simultaneous icECG recording. icECG was used for a beat-to-beat analysis of the ST-segment shift during reactive hyperemia immediately following balloon deflation. The time from coronary balloon deflation until the ST-segment shift reached 37% of its maximum level, i.e., icECG ST-segment shift remission time (τ-icECG in seconds), was obtained by an automatic algorithm. τ-icECG was tested against the simultaneously obtained reactive hyperemia FFR at a threshold of 0.80 as a reference parameter. From 120 patients, 139 icECGs (age, 68 ± 10 yr old) were analyzed. Receiver operating characteristic (ROC) analysis of τ-icECG for the detection of hemodynamically relevant coronary stenosis at an FFR of ≤0.80 was performed. The area under the ROC curve was equal to 0.621 (*P* = 0.0363) at an optimal τ-icECG threshold of 8 s (sensitivity, 61%; specificity, 67%). τ-icECG correlated inversely and linearly with FFR (*P* = 0.0327). This first proof-of-concept study demonstrates that τ-icECG, a measure of icECG ST segment-shift remission after a 1-min coronary artery balloon occlusion accurately detects hemodynamically relevant coronary artery stenosis according to FFR at a threshold of ≥8 s.

**NEW & NOTEWORTHY** Invasive hemodynamic measurements are recommended by the current cardiology guidelines to guide percutaneous coronary interventions in the setting of chronic coronary syndrome. However, those pressure-derived indices demonstrate several theoretical and practical limitations. Thus, this study demonstrates the accuracy of a novel, pathophysiology-driven approach using intracoronary ECG for the identification of hemodynamically relevant coronary lesions by quantitatively assessing myocardial ischemia remission.

## INTRODUCTION

Aside from acute coronary syndrome, where percutaneous coronary intervention (PCI) has been shown to improve outcomes (1), the number of patients with chronic coronary syndrome (CCS) is increasing. In these patients, PCI of hemodynamically relevant stenotic lesions causing myocardial ischemia is the standard treatment for symptomatic relief (2). Per definition, hemodynamically relevant coronary lesions are those inducing restriction to coronary blood flow beyond the autoregulatory myocardial microcirculation, which acts as a resistance-to-flow regulator and maintains resting blood flow over a broad range of mean arterial perfusion pressures between 50 and 150 mmHg (3). However, based on technical limitations to assess coronary blood flow (mL/min) or myocardial perfusion (mL/min/g myocardium) invasively, coronary stenoses are routinely assessed by structural visual angiographic estimates of diameter narrowing or by coronary pressure measurements up- and downstream the stenotic lesion. The latter is recommended by the ESC (2) and is based on its prognostic value derived from large randomized clinical trials (4). Given temporary maximal paralysis of the coronary microcirculation by a hyperemia-inducing substance such as adenosine, pressure is, in theory, directly and linearly related to coronary flow (5). Therefore, mean pressure during hyperemia downstream coronary stenosis relative to the upstream pressure [fractional flow reserve, FFR (6)] may provide an estimate of its restrictive effect on flow. However, this method depends on expensive pressure sensor angioplasty guidewires, on hyperemia-inducing substances and demonstrates several theoretical shortcomings. For instance, the requirement for a constant and maximal microcirculatory dilatation, essential for substituting flow by pressure, is challenged by decreasing FFR values at higher than standardized adenosine dosages (7). This indicates that the microcirculation is typically not fully dilated, and thus, implies that the equation linearly relating flow and pressure is not valid. Of note, this shortcoming also affects the drug-free alternative, i.e., instantaneous wave-free ratio (8).

Given these limitations, we explored a new method to assess hemodynamic coronary stenosis severity and focused on the dynamic behavior of myocardial ischemia immediately following a brief coronary balloon occlusion. The extent of myocardial ischemia, i.e., the mismatch between myocardial oxygen demand and supply depends on the duration of insufficient oxygen supply, the ischemic area at risk, myocardial oxygen consumption, and coronary collateral blood supply (9). Conversely, its remission depends on the velocity to offset the demand/supply mismatch, i.e., the postischemic hyperemic coronary blood flow. To assess this dynamic behavior, we used the intracoronary electrocardiogram (icECG). icECG is, in contrast to surface ECG, more time and site sensitive in detecting myocardial ischemia, owing to its close vicinity to the myocardial region of interest. Based on these characteristics, remission of myocardial ischemia after a brief coronary balloon occlusion can be observed in real time by a beat-to-beat analysis of the icECG signal (10, 11).

Thus, we hypothesized that icECG ST-segment shift remission time (subsequently called τ-icECG) as a measure of myocardial ischemia remission detects hemodynamically relevant coronary artery stenosis.

## METHODS

### Study Design

This was a retrospective observational single-center study in patients with CCS who participated in clinical trials of our research group, and therefore, underwent coronary angiography and hemodynamic measurements of coronary collateral function (collateral flow index, CFI) during an exactly 1-min proximal coronary artery balloon occlusion as started and terminated by stop clock (12). In this context, reactive hyperemia FFR immediately following coronary occlusion with simultaneous icECG recording was performed. Per patient, up to two measurements were carried out during an invasive examination. Patients with acute coronary syndrome, unstable cardiovascular conditions, severe aortic stenosis, paced cardiac rhythm, and severe renal and hepatic failure were excluded. Patients with left bundle branch block (LBBB), right bundle branch block (RBBB), atrioventricular block (AVB), atrial fibrillation (AF), and atrial flutter (AFL) were not excluded from the analysis.

CFI, the ratio between mean coronary occlusive to mean aortic pressure both subtracted by central venous pressure was obtained at the end of the above-mentioned, systematic 1-min coronary occlusion (13). So called sufficient collateral supply has been defined as CFI > 0.20–0.25, the fact of which is important as it is related to the absence of ischemic changes on icECG during a 1-min coronary balloon occlusion (14, 15). Patients with CFI higher than 0.20–0.25 were not excluded from this analysis.

All patients signed an informed consent form for further use of data in previous trials (10, 12), which have been conducted at the Department of Cardiology of the University Hospital Bern, and have been approved by the Ethics Committee of the Canton of Bern, Switzerland. The study was performed in collaboration with the University of Applied Sciences and Arts Northwestern, Switzerland. 

### cECG ST-Segment Remission Mean Lifetime

**Figure 1.**
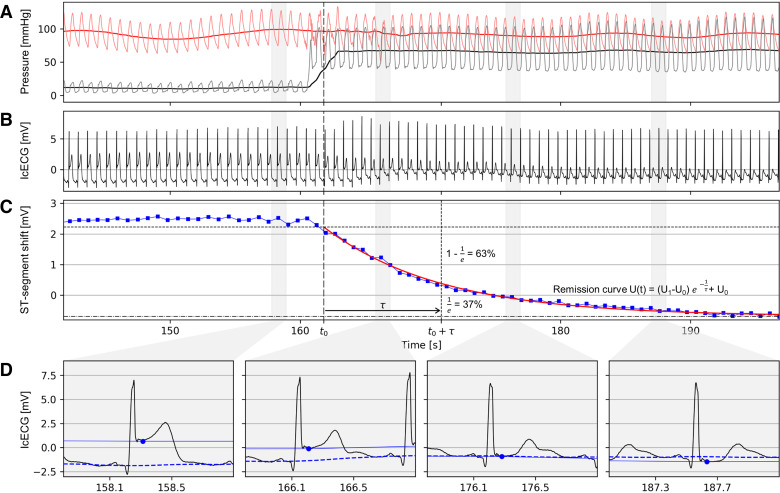
Algorithm analysis. *A*: simultaneous recordings of mean and phasic aortic (red signals) and coronary occlusive (black signals) pressure at the end of a coronary artery occlusion and after the balloon deflation. *B*: intracoronary electrocardiogram tracking. *C*: autonomous ST-segment tracking with the remission curve [U(*t*); red; plotted exponentially decaying curve for the calculation of icECG ST-segment shift remission time (τ-icECG)]. Vertical dashed line, *t*_0_ = end of the occlusion; horizontal dashed line, *U*1 = icECG ST-segment shift at the end of the occlusion; horizontal dashed and dotted line, *U*0 = initial ST-segment shift before the balloon occlusion. *D*: icECG QRS complexes at four different time points (recording time point illustrated by the gray shadow area).

icECG was recorded by connecting the pressure sensor wire (PressureWire TMX Guidewire, Abbott, Chicago, IL) positioned in the distal third of the coronary artery of interest to an alligator clamp, which was connected to chest lead V5 of the 12-lead surface ECG. The detailed acquisition of icECG has been described before (10). The complete icECG recording set immediately before, during the 1-min balloon occlusion, and throughout the postdeflation period of 120 s was used for a beat-to-beat analysis of the icECG ST-segment shift (Fig. 1). All quantitative icECG ST-segment shift measurements were performed by the autonomous algorithm described before (11).

The end of the coronary balloon occlusion t_0_ is automatically detected by analyzing the midpoint of the pressure rise curve in the distal coronary artery pressure (P_d_, mmHg). The rate of the icECG ST-segment remission is measured by τ-icECG, a characteristic time. τ-icECG is extracted by a model-based approach using the model of an exponentially decaying curve from the detected J-points (Fig. 1). The exponential decay model is:

$$U\left(t\right)\;=\;\left(U_{1}-U_{0}\right)e^{-\frac{t}{\tau }}+U_{0}$$

with the three parameters *U*_0_, *U*_1_, and τ (Fig. 1). The parameters are determined by a least squares optimization with integrated outlier (artifact) suppression. *U*_1_ indicates the ST-segment shift at the end of the occlusion period, *U*_0_ indicates the baseline of the ST-segment shift determined immediately before the balloon occlusion. Parameter τ corresponds to τ-icECG and provides a continuous measure of the ST-segment shift recovery mean life time, i.e., the time in seconds the ST-segment shift has decreased to 37% (= 1/*e* = mean life time) of the ST-segment shift developed during the occlusion (Fig. 1). Hence, a smaller τ-icECG value signifies a quicker recovery of the ST-segment shift, whereas a larger τ-icECG corresponds to a slower myocardial ischemia remission.

### Potential Confounders

τ-icECG as a measure of myocardial ischemia remission, i.e., the recovery of the supply/demand mismatch, has several potential confounders. On the demand side, the regional extent of myocardial ischemia, the duration of insufficient oxygen supply, myocardial oxygen consumption, and coronary collateral blood supply are potential confounders. Based on the study setting with a controlled, strictly 1-min proximal coronary balloon occlusion, only the last two factors are variable. On the supply side, i.e., the remission of myocardial ischemia, coronary blood flow, and the oxygen carrying capacity of the blood are influencing factors. Based on these considerations, heart rate, left ventricular ejection fraction (both myocardial oxygen consumption), left ventricular end-diastolic pressure (affecting the blood flow to the microcirculation), hematocrit (rheological factor), and hemoglobin (oxygen carrying capacity) are potential confounders.

### Hemodynamic Assessment of Coronary Artery Lesions

Coronary pressure measurements and all other study end points (hemoglobin level, left ventricular ejection fraction, or left ventricular end-diastolic pressure) were taken from the original study data. In brief, mean P_d_ and mean aortic pressure (P_ao_, mmHg) were continuously recorded for determining the reference parameter of the study, i.e., FFR. FFR (P_d_/P_ao_) was determined during reactive hyperemia and analyzed autonomously by the algorithm. Reactive hyperemia was induced by a strictly 1-min proximal coronary artery balloon occlusion in the vessel of interest (Fig. 1) immediately followed by angiography. This method of FFR measurement has been shown to be noninferior in its ability to detect relevant coronary stenosis compared with adenosine-induced FFR (16). Furthermore, it allowed the determination of CFI as a potential cofactor in relation to the extent of myocardial ischemia. Of note, myocardial ischemia during coronary artery occlusion develops over several heartbeats until a maximum ischemic steady state is reached, a process that has been described as more stable (17) and longer in duration (18) with postocclusion FFR than with adenosine-induced FFR. Importantly, Gloekler et al. (19) demonstrated that this method is safe regarding procedural and long-term complications.

Quantitative coronary angiography (QCA) was used to assess structural coronary artery lesion severity in two orthogonal planes, determining percent diameter narrowing (%S) with the guiding catheter for calibration.

### Statistical Analysis

For presentation purposes, the dataset was split in two groups according to hemodynamic stenosis relevance as determined by the reference method FFR at a threshold of ≤/>0.80. Continuous variables were compared by unpaired student’s *t* test or Mann–Whitney *U* test, as appropriate. The association between the primary study end-point τ-icECG and coronary pressure-derived parameters, as well as %S by QCA, was analyzed by linear regression analysis. Receiver operating characteristic, ROC analysis (area under the ROC curve, sensitivity, specificity) was used for diagnostic accuracy assessment of the primary study end-point τ-icECG in detecting hemodynamically relevant coronary stenosis using reactive hyperemia FFR as the primary reference method. The optimal cutoff value of τ-icECG was determined by the Youden index. A *P* value of <0.05 was considered statistically significant. All normally distributed, continuous variables are presented as means ± SD. Categorical variables are presented as fractions or percentages (%).

## RESULTS

### Patients Characteristics

During hemodynamic coronary measurements from 135 patients, 162 icECGs were included in this study. From each patient, up to two icECGs were analyzed per examination during reactive hyperemia after a 1-min proximal coronary balloon occlusion. Thirty-three (14%) had to be excluded from the analysis because of data corruption (i.e., incomplete icECG recordings or severe signal noise) in 12 (52%) cases, and algorithm limitations (i.e., severe icECG artifacts in the recordings) in 11 (48%) cases. Thus, the study population comprised 120 patients with 139 icECGs. At the time of study inclusion, 30 (25%) patients were female, and mean age of the study population was 68 ± 10 yr old.

### Association of τ-icECG with Stenosis Parameter

Mean τ-icECG was 11.5 ± 14.3 s with a range from 0.8 to 105 s. In the two study groups with FFR > 0.80 and ≤ 0.80, mean τ-icECG was 10.6 ± 14.2 s and 14.3 ± 14.5 s, respectively (*P* = 0.0363; Table 1). There was an inverse linear relation between τ-icECG and FFR: τ-icECG = 32 − 24 × FFR (*r*^2^ = 0.045, *P* = 0.0125). There was no statistically significant relation between τ-icECG and %S by QCA (*r*^2^ = 0.006, *P* = 0.361).

**Table 1.** Study end points

	Total	FFR > 0.80	FFR ≤ 0.80	*P* Value
Study measurements, *n*	139	106	33	
ECG baseline parameters				
Heart rhythm				0.44
Sinus rhythm, *n* (%)	131 (94)	99 (93)	32 (97)	
AF in history or in resting ECG before angiography, *n* (%)	8 (6)	7 (7)	1 (3)	
Bundle branch block (any)				0.29
No BBB, *n* (%)	115 (83)	88 (83)	31 (94)	
Left bundle branch block, *n* (%)	3 (2)	3 (3)	0	
Right bundle branch block, *n* (%)	10 (7)	8 (8)	2 (6)	
Left anterior hemiblock, *n* (%)	11 (8)	9 (9)	2 (6)	
Atrioventricular block, *n* (%)	30 (22)	26 (25)	4 (12)	0.13
Bifascicular block, *n* (%)	6 (4)	4 (4)	2 (6)	0.57
Coronary angiographic parameters				
PCI performed after study examination, *n* (%)	76 (55)	50 (47)	26 (79)	<0.001
Coronary artery measured				<0.001
Left anterior descending artery, *n* (%)	60 (43)	35 (33)	25 (76)	
Left circumflex coronary artery, *n* (%)	52 (37)	49 (47)	3 (9)	
Right coronary artery, *n* (%)	27 (19)	22 (21)	5 (15)	
Number of diseased vessels	1.8 ± 1.0	1.7 ± 1.0	2.3 ± 0.7	0.03
QCA, %diameter stenosis	43 ± 21	38 ± 19	61 ± 14	<0.001
QCA ≥ 50%, *n* (%)	43 (31)	20 (19)	23 (70)	<0.001
Coronary artery functional parameters				
Collateral flow index	0.101 ± 0.069	0.102 ± 0.071	0.098 ± 0.067	0.91
CFI ≥ 0.20, *n* (%)	9 (7)	7 (7)	2 (6)	0.91
CFI ≥ 0.25, *n* (%)	5 (4)	5 (5)	0	0.20
FFR, *n* (%)	0.855 ± 0.125	0.911 ± 0.063	0.675 ± 0.106	–
Angina pectoris during ostial COA, *n* (%)	97 (71)	68 (65)	29 (91)	0.006
Angina grade, 0–10	3.7 ± 2.6	3.6 ± 2.6	4.4 ± 2.6	0.42
Delta icECG ST-shift during coronary occlusion, mV	1.50 ± 0.99	1.58 ± 1.06	1.24 ± 0.62	0.16
τ-icECG, s	11.5 ± 14.3	10.6 ± 14.2	14.3 ± 14.5	0.036
τ-icECG ≥8 s, *n* (%)	57 (41)	37 (35)	20 (61)	0.009
Potential confounders				
Left ventricular end-diastolic pressure, mmHg	10 ± 6	10 ± 7	10 ± 6	0.98
Left ventricular ejection fraction, %	62 ± 10	61 ± 11	65 ± 8	0.11
Heart rate during coronary measurements, beats/min	85 ± 25	82 ± 24	98 ± 23	<0.001
Hemoglobin, g/L	139 ± 16	139 ± 15	138 ± 18	0.79
Hematocrit L/L	0.4 ± 0.0	0.4 ± 0.0	0.4 ± 0.0	0.80

Values are means ± SD or *n* (%). AF, atrial fibrillation; BBB, bundle branch block; COA, coronary artery occlusion; CFI, collateral flow index; ECG, electrocardiogram; FFR, fractional flow reserve; τ-icECG, intracoronary electrocardiogram ST-segment shift remission time; PCI, percutaneous coronary intervention; QCA, quantitative coronary angiography.

### Receiver-Operating Characteristic Analysis

**Figure 2.**
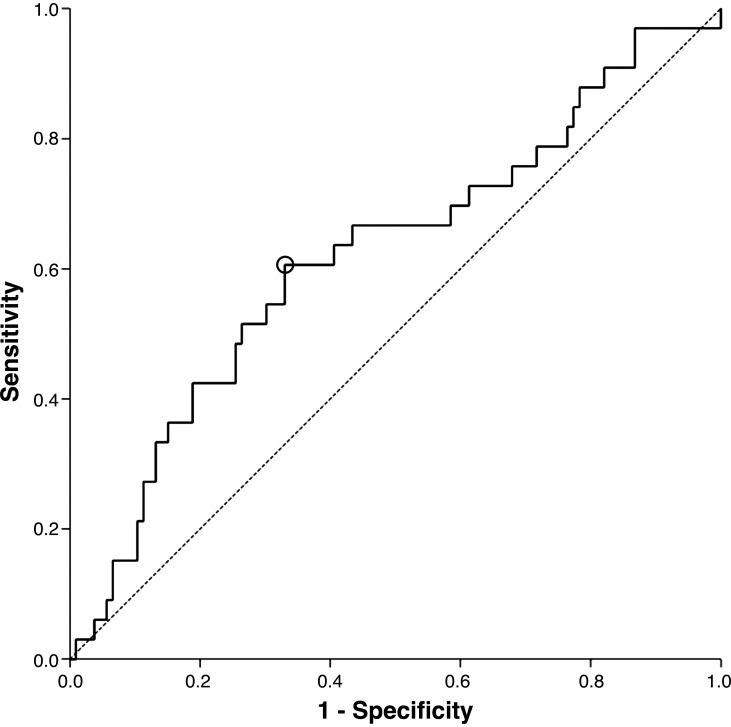
Receiver-operating curve analysis of intracoronary electrocardiogram ST-segment shift remission time (τ-icECG) values in the prediction of stenosis severity determined by a fractional flow reserve (FFR) value ≤ 0.8. Black circle marks the best cutoff point for τ-icECG according to the Youden index: 0.8 s sensitivity, 61%; specificity, 67%.

Using an FFR threshold of 0.80 as the reference for hemodynamic coronary stenosis relevance, ROC analysis of τ-icECG showed an area under the curve (AUC) of 0.621 (95% confidence interval, CI 0.507–0.735, *P* = 0.0363). Regarding the optimum cutoff for τ-icECG, ≥8 s detected hemodynamically relevant stenotic lesions according to FFR with a sensitivity of 61% and a specificity of 67% (Fig. 2).

### Potential Confounders

**Figure 3.**
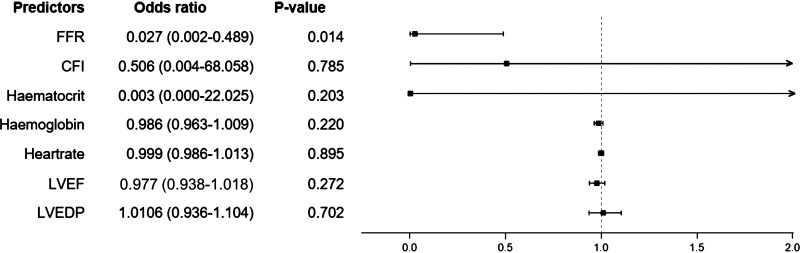
Forest plot of fractional flow reserve (FFR, mmHg/mmHg) and the potential confounders with an intracoronary electrocardiogram ST-segment shift remission time (τ-icECG) ≥ 8 s. CFI, collateral flow index (mmHg/mmHg); LVEF, left ventricular ejection fraction (%); LVEDP, left ventricular end-diastolic pressure (mmHg).

τ-icECG being potentially influenced by confounders, logistic regression revealed no association of τ-icECG with the following parameters (Fig. 3): CFI, hematocrit (Hct), hemoglobin (Hb), heart rate during hemodynamic measurement, left ventricular ejection fraction (LVEF), or left ventricular end-diastolic pressure (LVEDP). Reactive hyperemia FFR showed a significant association with τ-icECG (*P* = 0.0145; OR, 0.027; 95% CI, 0.002–0.489; Fig. 3).

## DISCUSSION

This retrospective proof-of-concept study assessed the diagnostic accuracy of the novel parameter τ-icECG obtained immediately after a standardized 1-min coronary balloon occlusion as a measure of myocardial ischemia remission for the detection of hemodynamically relevant coronary stenoses according to the reference method FFR. τ-icECG demonstrated a diagnostic accuracy of 62% with a threshold of ≥8 s in detecting reactive hyperemia FFR ≤ 0.80. Potential confounders of τ-icECG such as collateral function, heart rate, hemoglobin level, left ventricular ejection fraction, or left ventricular end-diastolic pressure did not show a significant association with τ-icECG because of a limited range of values (e.g., CFI, 0.101 ± 0.069; hemoglobin, 136 ± 16 g/L).

### Assessment of Coronary Stenosis Severity

The hemodynamic relevance of coronary lesions is uncovered when the restriction to coronary blood flow exceeds the compensatory autoregulatory threshold of the myocardial microcirculation, which itself aims at lowering circulatory resistance by vasodilatation. At rest, the stenosis resistance becomes predominant when epicardial coronary lesions surpass 80% in diameter narrowing, or 50% during hyperemia, i.e., maximally reduced microcirculatory resistance (20). Despite the well-documented basis using quantitative coronary angiography for revascularization decisions, physician-based visual assessment, known for its tendency to overestimate lesion severity, has remained the standard method for guiding revascularization (21). Hence, to minimize unnecessary PCI of erroneously assessed coronary lesions, hemodynamic stenosis parameters have been introduced. The first theoretical concept for the estimation of coronary flow impairment was proposed by Young et al. (22). This concept, however, was based on coronary flow measurement before and after revascularization and thus, not feasible to guide clinical decision-making (23). Nevertheless, the idea was pursued further and adjusted for clinical use by Pijls et al. (24), introducing FFR. FFR has then been validated against various exercise stress tests including dobutamine stress echocardiography and myocardial perfusion imaging (6). In a clinical outcome trial, angiography-plus-FFR-guided PCI demonstrated a benefit over angiography-guided PCI in terms of major adverse cardiac events for up to 2 yr with a lower number of revascularizations (25). Based on this clinical evidence, a class IA recommendation has been given for the application of FFR for hemodynamic assessment of coronary lesions in chronic coronary syndrome (2).

Despite the documented benefit of FFR-guided revascularization, it is important to emphasize that coronary pressure indices are not a true reference for myocardial ischemia. It has to be stressed that coronary blood flow, and not coronary pressure, is the source supplying energy to the myocardium (5). The theoretical pressure-flow relationship forming the basis of all pressure indices demonstrates several discrepancies compared with the actual pressure-flow relationship in humans (23). The prerequisite of a maximally paralyzed, dilated microcirculation has been challenged earlier by clinical trials using increasing dosages of pharmacological vasodilators (7, 8). Hence, in the absence of the condition of a constant and minimal resistance R to flow Q in Ohm’s law (i.e., Δ*P* = Q·R), P does not linearly change with Q (ΔP indicating the pressure drop across the coronary circulation, i.e., a further covariable of the Q-R relation) (26). The concept of FFR is, however, based on a proportional linear relationship between pressure and flow. Furthermore, as outlined by Stegehuis et al. (23), the pressure-flow relationship is represented by “a straight line but a nonzero pressure intercept.” Thus, a change in pressure is not directly proportional to a change in coronary flow. Also, the pressure gradient across a coronary lesion depends on multiple factors, such as diameter stenosis, stenosis length and morphology, coronary collateral function, coronary microcirculation, and magnitude of flow through the coronary artery (23, 27). Therefore, given high coronary blood flow, a localized atherosclerotic disease can induce a significant pressure drop without substantial impairment of coronary perfusion, and thus, no induction of myocardial ischemia (28). These considerations based on coronary blood flow as the critical determinant for myocardial supply might explain the results of the FAME-II trial, where 80% of patients with abnormal FFR values did not experience major cardiac events and over 60% did not require PCI throughout the 2-yr follow-up period (5).

### τ-icECG

In contrast to the surface ECG with its dependence on lead position and the patient’s habitus, icECG demonstrates higher sensitivity in detecting myocardial ischemia because of its close vicinity to the myocardial region of interest (11). Aside from its high sensitivity for detecting local myocardial ischemia, icECG exhibits also a good temporal sensitivity for dynamically changing ischemia (10). This temporal sensitivity is illustrated in Fig. 1 with icECG alterations receding within six heartbeats after restoration of the coronary blood flow, i.e., directly after the coronary balloon occlusion. icECG reflects the sum of all energy-dependent electrical processes in myocardial cells, and thus, directly the adequacy of myocardial perfusion. Hence, with the restoration of coronary blood flow after balloon deflation, immediate remission of the ST-segment shift sets in. From the myocardial cell’s perspective, the time until complete remission of the supply/demand mismatch, i.e., of myocardial ischemia, is directly dependent on the amount of substrate shortage and its recovery. This, in turn, is influenced by the determinants of ischemia, i.e., myocardial oxygen consumption, the viable myocardium at risk as well as collateral blood supply and factors influencing the mismatch recovery, i.e., coronary blood flow and blood oxygen capacity.

Directly after balloon deflation, i.e., in the postischemic period, maximal vasodilatation of the microcirculation is present to remove products of the metabolism (e.g., CO_2_), resulting in maximal hyperemic blood flow. Viable myocardium and insufficient collateral supply are required conditions for this behavior. The volume rate of maximal blood flow is then dependent on the hemodynamic relevance of the coronary lesion(s) and the coronary microcirculation. In parallel with the known inverse relationship between coronary stenosis severity and coronary blood flow, the extent of postischemic substrate delivery decreases as stenosis severity increases. Accordingly, τ-icECG as a measure of myocardial ischemia recovery and thus, measure of the offset of the substrate shortage, demonstrates the same inverse relationship with the degree of the coronary stenosis. Hence, τ-icECG represents a novel method for the detection of hemodynamically relevant coronary stenosis.

### Discrepancy Between τ-icECG and Fractional Flow Reserve

Similar to coronary flow reserve (CFR), τ-icECG captures both the coronary micro- and macrocirculation, thus providing an explanation for the discrepancy between τ-icECG and FFR in 38% of the cases. In this regard, the presented results are supported by comparisons in the literature between FFR and CFR showing similar disagreement between the two methods as demonstrated by positron emission tomography (27). Importantly and as outlined by Johnson et al. (27), this discrepancy reflects fundamental coronary pathophysiology aside from technical obstacles in obtaining intracoronary Doppler signals. However, in terms of clinical management, the just mentioned observation is relevant as only stenoses causing a reduction in flow and not necessarily in coronary pressure (see the autoregulatory range of up to 100 mmHg) warrant PCI (28).

### Limitations

The results of this study should be viewed in light of several limitations. First, it is limited by its retrospective nature, which resulted in the exclusion of a relevant percentage of patients because of data corruption. The retrospective design also played a role in the exclusion of 14% of the icECG recordings because of algorithm failures, primarily arising from severe signal artifacts or incomplete datasets. This dual limitation underscores the importance of improving algorithmic robustness and the necessity of prospective data collection for more accurate and comprehensive insights.

Second, logistic regression analysis did not yield statistically significant associations between τ-icECG and its covariables aside from the reference variable FFR, i.e., collateral function (CFI), heart rate, hemoglobin level, left ventricular ejection fraction, or left ventricular end-diastolic pressure despite their pathophysiological relevance. This may be in part explained by the present data’s relatively narrow range within normal limits, respectively by the presence of an almost exclusively insufficient collateral function (CFI > 0.20 in only 9 cases), which had no mitigating influence on myocardial ischemia.

Lastly, patients with pacemaker rhythm were excluded beforehand. Hence, applicability of this novel parameter for this population has yet to be validated.

On the other hand, diagnostic accuracy may be increased by conducting a prospective study. In addressing the technical limitation of data corruption and/or signal noise hampering, the analysis should be minimized by a prospective study with a detailed study protocol. Furthermore, addressing the currently existing class imbalance between the groups (i.e., FFR > 0.80; *n* = 106 vs. FFR ≤ 0.80, *n* = 33) can be expected to result in an enhanced diagnostic performance.

### Conclusion

This first proof-of-concept study shows that τ-icECG, a measure of icECG ST segment-shift remission after a 1-min coronary artery balloon occlusion accurately detects hemodynamically relevant coronary artery stenosis according to FFR at a threshold of ≥8 s.

## DATA AVAILABILITY

Data will be made available upon reasonable request.

## GRANTS

This work was supported by Schweizerischer Nationalfonds zur Förderung der Wissenschaftlichen Forschung Grants 32003B_184922/1 and 310030_172956/1 (to C.S.) and grants from Gottfried und Julia Bangerter-Rhyner-Stiftung (to A.K.-G. and M. R. B.) and Stiftung FHNW (to R.W.).

## DISCLOSURES

No conflicts of interest, financial or otherwise, are declared by the authors.

## AUTHOR CONTRIBUTIONS

M.R.B., A.K.-G., and C.S. conceived and designed research; M.R.B., A.K.-G., M.R., N.C., F.W., R.W., J.H., and C.S. performed experiments; M.R.B., A.K.-G, J.H., and C.S. analyzed data; M.R.B., A.K.-G., and C.S. interpreted results of experiments; M.R.B., A.K.-G., and C.S. prepared figures; M.R.B., A.K.-G., J.H., and C.S. drafted manuscript; M.R.B., A.K.-G., M.R., N.C., F.W., R.W., J.H., and C.S. edited and revised manuscript; M.R.B., A.K.-G., M.R., N.C., F.W., R.W., J.H., and C.S. approved final version of manuscript.
